# Dissemination Strategies for mHealth Apps: Systematic Review

**DOI:** 10.2196/50293

**Published:** 2024-01-05

**Authors:** Henri Claude Moungui, Hugues Clotaire Nana-Djeunga, Che Frankline Anyiang, Mireia Cano, Jose Antonio Ruiz Postigo, Carme Carrion

**Affiliations:** 1 Universitat Oberta de Catalunya Barcelona Spain; 2 Higher Institute for Scientific and Medical Research Yaounde Cameroon; 3 Texila American University Georgetown Guyana; 4 eHealth Lab Research Group eHealth Center & School of Health Sciences Universitat Oberta de Catalunya Barcelona Spain; 5 Prevention, Treatment and Care Unit Department of Control of Neglected Tropical Diseases World Health Organization Geneva Switzerland

**Keywords:** mobile health, mHealth, mobile health apps, mHealth apps, dissemination, marketing strategies, digital marketing, engagement, onboarding, systematic review, systematic, market, marketing, app, apps, adoption, consumer, mobile phone

## Abstract

**Background:**

Among the millions of mobile apps in existence, thousands fall under the category of mobile health (mHealth). Although the utility of mHealth apps has been demonstrated for disease diagnosis, treatment data management, and health promotion strategies, to be effective they must reach and be used by their target audience. An appropriate marketing strategy can ensure that apps reach potential users and potentially convert them to actual users. Such a strategy requires definitions of target end users, communication channels, and advertising content, as well as a timeline for effectively reaching and motivating end users to adopt and maintain engagement with the mHealth app.

**Objective:**

The aim of this study was to identify strategies and elements that ensure that end users adopt and remain engaged with mHealth apps.

**Methods:**

A systematic search of the PubMed, PsycINFO, Scopus, and CINAHL databases was conducted for suitable studies published between January 1, 2018, and September 30, 2022. Two researchers independently screened studies for inclusion, extracted data, and assessed the risk of bias. The main outcome was dissemination strategies for mHealth apps.

**Results:**

Of the 648 papers retrieved from the selected databases, only 10 (1.5%) met the inclusion criteria. The marketing strategies used in these studies to inform potential users of the existence of mHealth apps and motivate download included both paid and unpaid strategies and used various channels, including social media, emails, printed posters, and face-to-face communication. Most of the studies reported a combination of marketing concepts used to advertise their mHealth apps. Advertising messages included instructions on where and how to download and install the apps. In most of the studies (6/10, 60%), instructions were oriented toward how to use the apps and maintain engagement with a health intervention. The most frequently used paid marketing platform was Facebook Ads Manager (2/10, 20%). Advertising performance was influenced by many factors, including but not limited to advertising content. In 1 (10%) of the 10 studies, animated graphics generated the greatest number of clicks compared with other image types. The metrics used to assess marketing strategy effectiveness were number of downloads; nonuse rate; dropout rate; adherence rate; duration of app use; and app usability over days, weeks, or months. Additional indicators such as cost per click, cost per install, and clickthrough rate were mainly used to assess the cost-effectiveness of paid marketing campaigns.

**Conclusions:**

mHealth apps can be disseminated via paid and unpaid marketing strategies using various communication channels. The effects of these strategies are reflected in download numbers and user engagement with mHealth apps. Further research could provide guidance on a framework for disseminating mHealth apps and encouraging their routine use.

## Introduction

### Mobile Health Apps

Among the vast array of mobile apps currently available, health care apps serve various purposes, including disease diagnosis [1], health promotion, and disease prevention [2,3]. Such uses of mobile technology to provide patients with health care support or health service providers with technical support in a direct, low-cost, and engaging manner fall under the category of mobile health (mHealth) [4].

With approximately 200 new mHealth apps released every day, the number available now exceeds 300,000 [5]. One factor in this proliferation is the increasing use of mHealth technology by health service providers who not only seek advice from apps but also prescribe them to their patients [6]. In October 2020, Germany became the first country to cover the prescription costs of certain mHealth apps through statutory health insurance [7]. Moreover, because mHealth apps have the potential to replace a number of health provider tasks, it has been suggested that expertise in the use of mHealth-related technologies should be recognized as an essential competency for providers [1].

### Dissemination of mHealth Apps to Users

The amount of academic research on mHealth apps has also increased, particularly in the areas of usability, effectiveness, adoption, and assessment. However, the highly important aspects of dissemination and marketing are as yet underexamined. App marketing refers to measures aimed at making a mobile app better known and acquiring users (ie, generating app downloads) and, moreover, contacting users and encouraging them to reach a specified goal [8].

An mHealth app is not a stand-alone product that can work effectively without human interaction, which cannot take place if users are unaware that a certain app exists and is accessible. Such “human touches,” although extraneous to the app itself, can be crucial for promoting use [9].

As users are key actors in mHealth adoption, it is critical to understand how they navigate the various stages from app discovery to frequent use. Google has created such a model [10]. It consists of four key stages: (1) discover (users come across an app and download it to their device), (2) onboard (the process of first use and registration), (3) engage (users start using the app regularly), and (4) embed (the desired outcome as users view the app as “vital” to their lives). Only a small proportion of users currently reach the embedment stage with any app [10]; for instance, the literature is sparse regarding the long-term integration and penetration of mobile interventions within mental health and other support service settings [11].

Increasing the chances of an app achieving embedment requires understanding users and placing them at the core of mHealth services. This process would start with persona definitions: fictional archetypes of actual product users. A persona enables program designers to create high-quality programs that effectively meet user needs [2]. In the marketing world, this also means segmenting users and locating them on the marketing funnel, which is a visual representation of the different phases in a customer’s journey toward conversion and their relationship with a product. By segmenting customers based on where they are located in the funnel, marketers target these groups much more effectively [12].

Positive customer experiences and journeys rely on ensuring that the consumer sees value in an app as a channel for accessing products and services and as a 2-way platform for seamless interactions. Although marketing strategies play a crucial role during the early stages of the customer journey, they have been subjected to very little analysis [13].

### Marketing of Health Apps

A successful marketing strategy can ensure that an mHealth app reaches potential users and ease the adoption process. Such a strategy would clearly define target end users; determine the appropriate communication channels, content, and timelines to effectively reach users; and market the app as an attractive product, encouraging people to download it and become regular users. The strategy would include a mix of activities, depending upon the type of app and upon the stage of the launch period (from before the launch to after the launch), including email marketing, targeted advertising, and social media promotion [14].

Marketing services have evolved alongside information and communication technologies. In turn, digital marketing has provided a series of customized platforms for communicating with specific stakeholders using computers, smartphones, and tablet computers [15]. These channels enable information to be gathered and include websites as well as various social media platforms such as Facebook, YouTube, X (the platform formerly known as Twitter), Pinterest, TikTok, and LinkedIn. Traditional marketing also remains an option, with products being promoted on radio and television channels, as well as via printed posters in public spaces, flyers, and face-to-face conversations [16].

The cost of promoting an app will depend upon where money is spent, and those promotional activities that do not cost money will demand time. Although potential customers may be offered incentives such as money or supplemental products, the marketer or marketing firm involved in digital marketing can be offered a fee per click, download, or install. A small pilot trial of activities that cost money is recommended to assess results [14].

Specialized services are available for driving digital campaigns; for example, Facebook Ads Manager is a paid service that oversees paid digital marketing campaigns across the Facebook platform. Google Universal App Campaigns (UAC) is another paid service that promotes mobile apps by distributing marketing messages across Google formats and networks, such as the first page of applicable Google search results and small banner advertisements on relevant YouTube channels [17]. As Google shares information among platforms, including Google Display Network, YouTube, and Google Play Store, Google UAC can capture the number of Google-driven impressions, clicks, and installs on Android devices.

The effectiveness of a marketing strategy can be observed not only through the number of app downloads but also by the effects of users interacting with the app. Referred to as mobile app engagement, this is defined by a variety of operational metrics, such as the number of log-ins, the number of days of use, the number of pages visited, and the number of tasks or modules completed [18]. Another gauge of marketing effectiveness is user onboarding. In the context of mobile apps, user onboarding is the process of providing instructions and highlighting key benefits and features via a set of example screens when the user first launches the app [19].

### Objectives

The aim of this study was to review existing evidence on strategies and elements relevant to how the dissemination of mHealth apps is currently carried out and how these elements contribute to encourage user onboarding and engagement with mHealth apps.

## Methods

### Overview

This study was carried out following the PRISMA (Preferred Reporting Items for Systematic Reviews and Meta-Analyses) guidelines [20] (Multimedia Appendix 1). The protocol of this review was registered with PROSPERO (CRD42022352369) [21].

### Eligibility Criteria

Eligible sources were academic papers. All cohort studies, cross-sectional studies, and randomized controlled trials reporting on the dissemination and marketing of an mHealth app were included. The outcome expected from included studies was strategies and elements relevant to how the dissemination of mHealth apps is currently carried out and how these elements contribute to encourage user onboarding and engagement with mHealth apps.

Editorials, letters to the editor, scoping reviews, systematic reviews, meta-analyses, conference abstracts, commentaries, essays, book chapters, and study protocols were excluded, as were studies with any other study design (eg, bibliometric analysis, modeling study, systematic or web search or review of apps, landscape analysis, and scorecard analysis). We also excluded studies with participants aged <18 years and those not reporting the expected outcome. Search languages were limited to French and English.

### Information Sources and Search Strategy

Searches were conducted on PubMed, PsycINFO, Scopus, and CINAHL databases over the 5-year period from January 1, 2018, to September 30, 2022 (refer to Multimedia Appendix 2 for the search strategy). The following search terms were used individually or combined according to Medical Subject Headings terms: “apps,” “mHealth,” “marketing,” “promotion,” and “dissemination.” Moreover, we conducted searches on JMIR and mHealth journals and cross-checked the reference lists of the selected studies to locate additional studies that met the inclusion criteria. The main outcome was dissemination strategies for mHealth apps.

### Study Selection and Data Collection Process

All retrieved studies were imported into Rayyan (Rayyan Systems Inc) [22] and duplicate records eliminated. Screening consisted of blind peer review by 2 independent investigators. Any conflict was resolved through discussion or the adjudication of a third investigator.

## Results

### Selection of Studies

**Figure 1:**
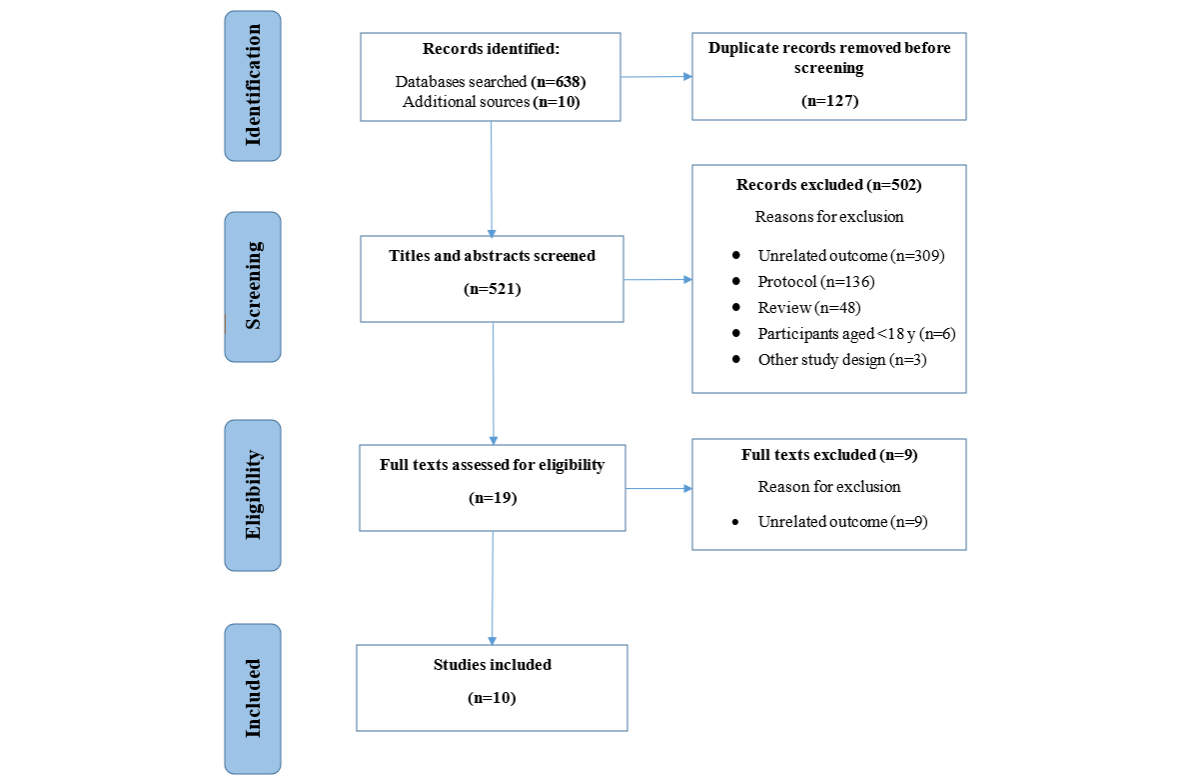
PRISMA (Preferred Reporting Items for Systematic Reviews and Meta-Analyses) flow diagram of the paper selection procedure.

We identified 638 records through database searches (PubMed: n=215, 33.7%; PsycINFO: n=60, 9.4%; Scopus: n=283, 44.4%; and CINAHL: n=80, 12.5%) and 10 records through JMIR and mHealth journal searches. Of the total 648 records, 127 (19.6%) duplicates were removed. Next, of the remaining 521 articles, 502 (96.4%) were removed after title and abstract screening. The reasons for exclusion were unrelated outcome or outcome other than the subject of our review (309/502, 61.6%), study protocol (136/502, 27.1%), published review (48/502, 9.6%), study participants aged <18 years (6/502, 1.2%), and other study design (3/502, 0.6%). We then assessed the remaining 19 full texts for eligibility and excluded 9 (47%) for unrelated outcomes; thus, 10 (53%) papers were included in this review. A PRISMA-compliant flow diagram [20] of the paper selection process is shown in Figure 1. The characteristics of the studies selected are summarized in Table 1.

**Table 1:** Characteristics of the selected studies.

Authors, year; country	Population	Intervention	Outcomes	Design	Quality	Limitations
Kvedarienė et al [23], 2019; Lithuania	N=149 Sex: 55% female Age: 18-60 (mean 37.2, SD 10.4) years Other details: Lithuanians with allergic rhinitis and asthma	Monitoring of allergic rhinitis and asthma in real life in Lithuanian MASK‐air app users	High app engagement User retention rate was 107 days of use/user Patients are satisfied with the app overall	Longitudinal study	Low	Selection bias: patients recruited from an allergy clinic
Buss et al [24], 2022; Australia	N=46 Sex: 50% female Age: ≥45 (median 62, IQR 56-67) years Other details: residing in Australia	Test the feasibility of an app-based intervention for cardiovascular and diabetes risk awareness and prevention	The app scored the highest for the information section and the lowest for the engagement section of the scale Nonuse and dropouts were too high, and adherence was too low to consider the intervention in its current form feasible Asking people aged ≥45 years to download the app and expect them to use it over 3 months without additional interaction was not feasible	Nonrandomized controlled trial (cohort study)	Low	Small sample size Nonuse and dropouts too high, adherence too low
Arshanapally et al [17], 2022; United States	N=NI^a^ Sex: NI Age: 18-45 years Other details: parents with children aged <5 years, speaking English or Spanish	Investigate the outcomes of a paid digital marketing campaign to promote an mHealth^b^ app about parent-engaged developmental monitoring	Paid digital marketing can be an effective strategy to promote mHealth apps targeting parents of young children Google-driven marketing messages in English had a higher clickthrough rate than those in Spanish	Implementation study	Low	No relevant limitations
Resnick et al [25], 2021; United States	N=41 Sex: 76% female Age: ≥18 (mean 51, SD 12) years Other details: non-Hispanic Black patients, speaking English	Assess the usability, acceptability, and user engagement of the Healthier Together mobile app	The app strongly engaged participants, with promising results on participants’ knowledge of cancer prevention behaviors and success in achieving their cancer prevention behavioral goals	Mixed methods intervention	Low	Small sample size Nonprobabilistic purposive sample of non-Hispanic Black patients at 2 internal medicine primary care clinics Participants were incentivized US $40 to complete the baseline, in-person, 40-month enrollment process and interview and US $60 for completing the 45-month exit telephone interview
Zlotorzynska et al [26], 2021; United States	N=NI Sex: 0% female Age: 18-24 years Other details: YMSM^c^	Paid web-based recruitment campaign to recruit HIV-negative or unknown status YMSM for 4 randomized controlled trials of mHealth HIV prevention interventions	Instagram advertisements yielded the highest proportions of eligible contacts who were racial or ethnic minority individuals and aged <18 years	Randomized controlled trials	Moderate	Participants offered incentive to enroll in study
Rajani et al [27], 2021; United Kingdom	N=154 Sex: 38.8% female Age: 18-65 years Other details: smokers	Examine the association among perceived usefulness, perceived ease of use, and frequency of use of gamification features embedded in smoking cessation apps on self-efficacy and motivation to quit smoking	The use of the apps was associated with increased self-efficacy and motivation-to-quit levels 4 weeks after app use compared with baseline Perceived frequency of use of gamification features was associated with an increase in self-efficacy and motivation to quit Higher baseline self-efficacy and motivation to quit were both associated with smaller increases in self-efficacy and motivation-to-quit levels 4 weeks after using the mobile apps compared with preapp use	Observational study	Low	Participants incentivized
Roberts et al [28], 2019; United Kingdom	N=32 Sex: 68.8% female Age: ≥18 (mean 60, SD 11; range 37-78) years Other details: diagnosed with breast, prostate, or colorectal cancer	To seek opinions of survivors of breast, prostate, and colorectal cancer regarding using apps to promote PA^d^	Multiple factors affect engagement with PA apps, and this is highly personalized Apps that promote walking are the most appealing for survivors of cancer PA apps should be integrated into cancer care	Cross-sectional study	Low	Small sample Participants were offered a £10 (US $12.7) voucher as an incentive for completion of study, and costs were reimbursed if asked to install an app that was not free to download
Bidargaddi et al [29], 2018; United States and Australia	N=1255 Sex: NI Age: NI Other details: NI	To study the effect of time-varying push notifications on engagement in self-monitoring activity	Pushing a notification with a tailored health message affects near-time proximal engagement with the self-monitoring activity in the app	Microrandomized trial design	High	No relevant limitations
Hui et al [30], 2018; United States	N=101 Sex: 87.1% female Age: ≥16 years Other details: patients with active asthma	The impact of different recruitment strategies and app features on adoption and continued use	Adherence was dependent upon motivation derived from a sense that the health care professional and researcher were interested in the results and that using an app to support their self-management could improve their asthma control	Cohort study	Low	Selection bias: participants recruited at clinics
Haile et al [31], 2018; Egypt, Ghana, India, and Jordan	N=NI Sex: 100% female Age: ≥18 years Other details: NI	Market tests of the CycleBeads app in 7 countries	Social media campaigns proved to be an easy low-cost approach to advertising the CycleBeads app	In-app microsurveys	Low	No relevant limitations

^a^NI: no information.

^b^mHealth: mobile health.

^c^YMSM: young men who have sex with men.

^d^PA: physical activity.

Of the 10 selected studies, 6 (60%) were observational studies (longitudinal, cohort, implementation study, mixed methods, and cross-sectional), 1 (10%) reported on 4 randomized controlled trials [26], 1 (10%) reported on in-app microsurveys [31], and 1 (10%) reported on a microrandomized trial [29].

According to the Scottish Intercollegiate Guidelines Network (SIGN) [32] criteria, 8 (80%) of the 10 studies were of low quality, 1 (10%) of moderate quality, and 1 (10%) of high quality. A low-quality rating resulted from a small sample size, the study design (mostly observational), or possible selection bias. A summary of the design, quality, and limitations of the included studies can be found in Table 1.

The selected 10 studies covered 8 countries—Lithuania, the United Kingdom, Australia, the United States, Egypt, Ghana, India, and Jordan—with 50% (5/10) of the studies conducted in the United States and 20% (2/10) in the United Kingdom.

The number of participants in the selected studies ranged from 32 to 1255. Of the 10 studies, 3 (30%) that recruited participants through social media and used impressions and clicks as a proxy measure of their number did not state the number of participants. In 6 (60%) of the 10 studies, 50% to 100% of the participants were female; sex information was not given in 2 (20%) of the 10 papers, 1 (10%) study targeted adolescent and young male individuals, and 1 (10%) targeted female individuals only.

### Objectives of the Marketing Strategy

In each of the studies reviewed, we looked for the objective of the marketing strategy implemented through the lens of the Google model of mobile app user journey (discover, onboard, engage, and embed) [10] (Table 2). We found that 60% (6/10) of the studies aimed to drive people to discover their mHealth apps (come across the apps and download them), get onboard (start using the apps), and stay engaged by using the apps for a couple of weeks or months. Marketing strategy in 30% (3/10) of the studies targeted the discover and onboard stages, whereas 1 (10%) of the 10 studies aimed at the engage stage only.

**Table 2:** Summarized results of reviewed studies.

Authors, year	Channels	Procedures of each dissemination strategy: How was it done?	Effects and results of each dissemination strategy	Objective with regard to the Google model for users’ journey with apps
Kvedarienė et al [23], 2019	Face-to-face	Health personnel trained patients how to use the app at a clinic	The duration of app use in patients ranged from 1 to 680 (median 54, IQR 23-151) days There was an increased frequency when the reported days were >200 (18%) Adherence to mobile apps was higher when the app was promoted by physicians and when the users were taught how to use it	Discover, onboard, and engage
Buss et al [24], 2022	Email	Participants received an email and a user guide that included instructions to download an app from the app store on their mobile phones and then use it for 3 months Researchers encouraged regular use If they had questions or technical issues, participants could get in touch with researchers via email	Of the 46 participants, 20 (43%) never used the app, and 15 (33%) dropped out Adherence to app use (using the app at least once/week over 3 months) was 17% (8/46) The median time between the first and last app use was 54 days The research team did not actively engage with participants early in the study to verify that all participants could install the app The intervention did not involve direct contact with health care professionals The app did not contain enough interactive features	Discover, onboard, and engage
Arshanapally et al [17], 2022	Social media	Google- or Facebook-driven marketing message: when a user clicked on a marketing message, they were directed to either the Google Play Store or the Apple App Store, depending upon device type Google Universal App Campaigns distributed marketing messages across several Google formats and networks Facebook Ads Manager distributed marketing messages across the Facebook platform	The Google-driven marketing messages garnered a total of 4,879,722 impressions and 73,956 clicks (clickthrough rate: 1.52%); from these clicks, there were 13,707 installs of the app on Google Play Store (18.53% install rate); the overall cost/install was US $0.93 The Facebook-driven marketing messages garnered a total of 2,434,320 impressions and 44,698 clicks (clickthrough rate: 1.84%); the average cost/install for Facebook could not be calculated because of the limitation of collecting Facebook-driven installs data Animated graphics generated the greatest number of clicks among both English and Spanish audiences on Facebook when compared with other types of images	Discover, onboard, and engage
Resnick et al [25], 2021	Face-to-face	In-person information about the app, followed by installation of the mobile app on the participant’s mobile phone and instructions on how to select a goal, choose share settings, and invite other social ties Weekly SMS text messages reminding participants to check in, share goal progress, and invite relatives	Of the 171 participants, 41 (24%) downloaded the app Engagement with the app (mean number of check-ins/participant out of 8 possible weekly check-ins during the study period) was 5.7 Of the 41 participants, 31 (76%) checked in during at least 4 of the 8 weeks The mean System Usability Scale score was 87 (SD 12; median 90, IQR 78-95)	Discover, onboard, and engage
Zlotorzynska et al [26], 2021	Advertisements on Grindr, Snapchat, Instagram, and Facebook	Advertising was purchased on Facebook, Instagram, Snapchat, Twitter, and Grindr; users who clicked on banner advertisements were taken directly to a study-specific eligibility screener and, if eligible, were asked to provide contact information for follow-up by respective study site staff The Facebook Ads Manager proprietary algorithm allocated the distribution of advertisement placements (news feed, right-hand column, and Instagram feed and stories) that would provide the best performance Advertising copy was written to give a very brief description of the study, identify the organization conducting study recruitment, mention the study incentive, and provide a call to action for potential participants seeking to learn more Snapchat advertisements were created using Snap Publisher and were in the form of short videos up to 10 seconds long; these videos rotated through various stock photo images and superimposed text Instagram placements were used to test advertisement performance Images used in advertisements included stock photos, which were either purchased from web-based stock photo vendors (eg, Shutterstock) or accessed through Facebook’s free stock photo catalog integrated within the Facebook Ads Manager Recruitment through various in-person and community outreach efforts included posting flyers, tabling at community events, reaching out to past study participants, and recruiting through clinics serving youth	Grindr and Snapchat advertisements produced the highest clickthrough rate compared with Facebook advertisements; these advertisements had the lowest proportions of users who initiated eligibility screeners Facebook advertisements yielded the lowest cost/eligible contact, whereas advertisements on Twitter had the highest Facebook or Instagram advertisements had much higher rates of screening and ultimately yielded much lower costs/eligible participant The cost/eligible participant was markedly higher for the Instagram advertisements campaigns	Discover and onboard
Rajani et al [27], 2021	Social media and paper posters	Participants were recruited via social media, and posters were displayed in public places in London, United Kingdom Participants were provided instructions on the internet on how to download and start using the app	Of the 138 participants who installed the app, 116 (84.1%) completed all 4 weeks of the study A 1-point increase in the average perceived frequency of gamification features was statistically significantly associated with a 3.35-point increase in self-efficacy from baseline to study end (β=3.35, 95% CI 0.31-6.40)	Discover and onboard
Roberts et al [28], 2019	Face-to-face, paper posters, email, and social media	Participants recruited via advertisements within community-based cancer support groups (either by verbal descriptions from group leaders at meetings or via posters, flyers, and email mailing lists), Facebook cancer support groups, and charitable organizations Participant randomly assigned to download 2 of 4 apps (Human, The Walk, The Johnson & Johnson Official 7 Minute Workout, and Gorilla Workout) Guidance in downloading and installing each app was provided, if required; participants were asked to spend approximately 2 consecutive weeks using the apps	Of the 40 participants, 32 (80%) completed the study (dropout rate: 20%) Reasons for dropping out were lack of time, family circumstances (eg, bereavement), and not wanting to update their smartphone’s operating system or register credit card details with Google Play Factors affecting engagement included participants’ perceptions of the advantages and disadvantages of using apps to support physical activity, the relevance of the app to the user, the quality of the app, and the behavior change techniques used to promote physical activity	Discover, onboard, and engage
Bidargaddi et al [29], 2018	Push notifications	Push notifications were sent at 1 of 6 chosen time points throughout the day, and a user could either receive or not receive a push notification at a chosen time point At each considered time point, users were randomized to either receive or not receive a push notification containing a tailored health message with a 50% probability; once a time point was considered, the user was then considered unavailable for the remainder of the day To mitigate the risk of users either turning off notifications or deleting the app owing to receiving too many push notifications, users were classified as either “available” or “unavailable” at each time point, and only those time points when users were “available” were considered for the push notification decision; several rules were applied to determine availability	Sending a push notification containing a tailored health message was associated with greater engagement in a mobile health app The effect of the pushed notifications is sustained over time; push notifications containing tailored health messages can attenuate the rate at which users disengage Users who used the app less frequently were “unavailable” to receive push notifications on a greater number of days than those who used the app more often	Engage
Hui et al [30], 2018	Social media and face-to-face	Researchers sent letters inviting people to use the app for up to 3 months Practice nurses approached adults with active asthma to try out a prototype app The app was also advertised via social media (Facebook and Twitter [now known as X]) Nurses were not expected to teach patients to download and use the app Technological support was provided by the research team and the app developer The messages on social media included brief information and a link to information for patients on how to download the app	A total of 300 patients received information on the app and study from Facebook, Twitter, or organic searches Only 135 patients downloaded the app, with 111 (82.2%) registering an account on the app Social media attracted 87 users, but only 15 (17%) used the app for 30 days A total of 24 patients were recruited, and 13 (54%) continued for 30 days Successful adoption was dependent upon the ease of downloading and sufficient motivation Some patients needed technological assistance with downloading the app and starting to use the features	Discover, onboard, and engage
Haile et al [31], 2018	Social media and face-to-face	Series of culturally appropriate Facebook campaigns for each country of interest	Within 10 months after advertising, there were 356,520 app downloads; the cost/download (paid by the advertiser) was lowest in Nigeria at US $0.17, followed by Egypt (US $0.26), Ghana (US $0.27), India (US $0.30), Jordan (US $0.36), Kenya (US $0.41), and Rwanda (US $0.69) Most of the app users were aged 20-29 years and married or in exclusive relationships	Discover and onboard

### Channels and Procedures of Each mHealth App Dissemination Strategy

As shown in Table 2, mHealth app promotion was conducted through various channels, including paper posters, face-to-face communications, email, and social media. The most reported channels were face-to-face [23,25,28,30,31] and social media [17,27,28,30,31], both of which appeared in 50% (5/10) of the studies. Messages were intended to inform people about the existence of the app, what it does and the benefits of using it, where to find the downloading link, downloading instructions, and information on how to use the app. Messages through these channels used text, voice-overs, and short videos. Messages on social media and email included a shortened URL to download the app. Face-to-face interactions [23,25,28,30,31] provided the opportunity for training support, including demonstrating app features, installing and using the app, and solving technical issues.

Google Play Store and Apple App Store were the main web repositories to which potential users were redirected for download. In addition, the social media pages of various organizations, such as the Facebook pages of cancer support groups [28], were also used to promote apps and provide download links.

### Effects of Dissemination Strategies

Advertising performance was influenced by many factors, including but not limited to advertising content and creativity, competing advertisements within platforms, and emerging platforms that attracted users to new digital spaces [26]. The reviewed papers revealed that social media advertisements attracted substantial downloads over a short period with a large number of impressions [13] and prompted patients to respond to invitations to use the app. However, without the motivation provided by a trusted professional, users quickly ceased their use of the app; a dual promotion strategy was then used to increase app adoption: using social media for its reach and ease of response as well as promoting personal invitations from trusted health care professionals [25].

In 1 (10%) of the 10 studies, embedded push notifications were sent to users to keep them engaged with the app [24]. This effect was sustained over time, and push notifications containing tailored health messages reduced the rate at which users disengaged. The timing of notifications was a key factor, with the results suggesting that users were more likely to engage with an app within 24 hours when push notifications were sent at midday on a weekend. Animated graphics generated a greater number of clicks than other image types [17].

The success of mHealth app dissemination strategies in these studies was measured in terms of adherence, app use, the duration of app use, impressions, clicks, app downloads, and installs. Marketing messages with simple direct calls to action, such as “Track your child’s development,” generated high clickthrough rates (CTRs) [17].

### Marketing Messages and Content of Communications

In some cases, promotional messages included questions prompting people to download and test the app, such as “Would you like to test an app that helps you manage your asthma better? [shortened URL] Need a helping hand to manage your asthma? Try our new app [shortened URL]” [30]. One study included the inquiring headline “Ever think of testing at home?” and explanatory body text: “Fenway Health is looking for young men to help test out a mobile app to support sexual health” [26].

In a study promoting an mHealth app about parent-engaged developmental monitoring [17], marketing messages with animated graphic interchange format (GIF) images as well as images of younger children performed higher than messages without GIF images and images of older children.

### Paid or Nonpaid Marketing Campaigns

The paid marketing campaign platforms that were used included Facebook Ads Manager and Google UAC; in addition, advertisements were placed on Grindr, Snapchat, and Instagram [17,26,31]. In 50% (5/10) of the studies, participants were offered incentives to participate [24-28]. Of these 5 studies, 4 (80%) were conducted in the United States and 1 (20%) in Australia. No study reported that paid marketing resulted in higher app adoption than unpaid campaigns.

### Key Indicators of mHealth App Marketing Strategies

Various metrics were used in the reviewed studies to assess mHealth app marketing strategies. Key indicators included nonuse rate, dropout rate, and adherence rate, as well as median time between first and last app use, which was generally measured in days or weeks. Nonuse rate was defined as the proportion of participants who never used the app [24], and the duration of use was assessed by determining the reported number of days of use [23]. The longest duration of app use was 680 days [23].

Dropout rate was defined as the proportion of participants who completely stopped using the app at least 14 days before they received an invitation to complete the end-of-study survey. Adherence rate was defined as the proportion of participants who used the app at least once every week over 3 months of the study [24].

Specific indicators for paid marketing strategies were cost per click (CPC), defined as the amount of money spent per click secured in each advertisement campaign or advertisement set; CTR, the number of clicks divided by impressions; and cost per impression [26]. Impression is the number of times an advertisement is viewed by a user on an advertising platform. For advertisements seen multiple times by the same user, each view is counted as an impression. Additional indicators were the number and percentage of clicks that took people through to the eligibility screener and of those who were eligible for the study [26].

### People Implementing the Marketing Processes

In the studies reviewed, we found that promotional messages were mainly disseminated by researchers, trained nurses, and other health practitioners. Technological support was only provided by the research team and app developers. In some cases, recently enrolled patients contributed to promoting the app by sending download invitations to their family and friends [25].

### Marketing Concepts Reported in the Reviewed Studies

Inspired by a book on how to successfully distribute apps [12], we screened the included studies to identify the marketing concepts that were used to promote mHealth apps in these studies (Table 3).

**Table 3:** Marketing concepts [12] reported in the reviewed studies.

Authors, year	Incentivization^a^	Personalization^b^	Mobile app attribution^c^	Loyalty marketing^d^	Remarketing or retargeting^e^	A/B testing^f^	Programmatic marketing^g^	Predictive marketing^h^	Thought-leadership marketing^i^	Content marketing^j^	Behavioral marketing^k^
Kvedarienė et al [23], 2019^l^		✓									✓
Buss et al [24], 2022^l^	✓	✓									
Arshanapally et al [17], 2022^l^			✓			✓					✓
Resnick et al [25], 2021^l^	✓	✓	✓								✓
Zlotorzynska et al [26], 2021^l^	✓	✓	✓			✓				✓	
Rajani et al [27], 2021^m^	✓		✓								
Roberts et al [28], 2019^m^	✓									✓	
Bidargaddi et al [29], 2018^l^		✓	✓	✓				✓			✓
Hui et al [30], 2018^m^			✓						✓	✓	
Haile et al [31], 2018^m^			✓							✓	

^a^The incentivized model is the strategy of making a product, program, or other offering more attractive to customers by offering an incentive in exchange for buying or participating. In the app business, incentivization is normally used to quickly amass app installs. Of the 10 studies, half of the studies (n=5, 50%) used incentivization.

^b^Personalization involves customizing the timing and content of marketing messages to the target user based on their preferences, habits, and behavior patterns. Personalized messages refer to every user by name and entice the user to become more engaged with an app with the right kind of incentive based on their characteristics, such as age, gender, location, profession, and financial segment. Half of the studies (5/10, 50%) used personalization.

^c^Mobile app attribution is the process of recording and measuring the actions of app users, such as installs, level completions, in-app purchases, and other milestones. Mobile app attribution is essential to app marketing because it helps produce the data that are gathered and analyzed to measure how well marketing campaigns are working. The majority of the studies (7/10, 70%) used mobile app attribution.

^d^Loyalty marketing is a marketing strategy that focuses on nurturing existing customers rather than acquiring new ones. Only 1 (10%) of the 10 studies used loyalty marketing.

^e^Remarketing or retargeting targets every individual who has come into contact with the product but has not converted or who converted but later abandoned the app. It allows marketers to reconnect with these categories of users and “bring them back” or increase the time they spend engaging with the app. None of the studies used remarketing or retargeting.

^f^A/B testing involves the use of several versions of the same advertisement distributed to different groups with different designs, color coding, calls to action, and message content to determine which version produces the highest conversion rate. Of the 10 studies, only 2 (20%) used A/B testing.

^g^Programmatic marketing is the automated algorithm-based real-time buying and selling of advertising space through a bidding system, with the aim of reaching the right customers at the right time. None of the studies used programmatic marketing.

^h^Predictive marketing involves using data science based on customer behavior and habits to make smarter marketing decisions. By gathering and analyzing data about user behavior and identifying patterns, marketers can make forecasts about user behavior and make informed decisions about the likelihood of the success of their marketing content and offerings. Of the 10 studies, only 1 (10%) used predictive marketing.

^i^Thought-leadership marketing is the process of positioning a company as a leader in a specific domain by supplying customers with top-quality information. Only 1 (10%) of the 10 studies used thought-leadership marketing.

^j^Content marketing is a marketing strategy that involves producing content that potential customers find useful, valuable, and relevant. Content marketing is highly effective at building a loyal user base and converting leads into customers. More than one-third of the studies (4/10, 40%) used content marketing.

^k^Behavioral marketing involves segmenting the app’s user base based on user behavior with the aim of refining the marketing strategy and more effectively targeting users. More than one-third of the studies (4/10, 40%) used behavioral marketing.

^l^These studies (n=6, 60%) used single-channel marketing, which involves reaching users through a single channel, eg, Facebook advertisements.

^m^These studies (n=4, 40%) used multichannel marketing, which involves >1 channel, as opposed to an omni-channel marketing campaign, which attempts to reach users through all available channels.

In summary, most of the studies (7/10, 70%) reported using a combination of marketing concepts to advertise their mHealth apps. The most used concept was mobile app attribution (7/10, 70%). Mobile app attribution is essential to app marketing because it helps produce the data that are gathered and analyzed to measure how well marketing campaigns are working [12]. Furthermore, 40% (4/10) of the studies reported using at least 2 channels to market their mHealth apps.

## Discussion

### Principal Findings

We found that the marketing strategies used in almost all included studies (9/10, 90%) were aiming to drive at least app discovery and onboarding, with more than half (6/10, 60%) also targeting user engagement to mHealth apps. Social media, emails, television or radio, posters or flyers, and face-to-face communications were all used in the reviewed studies to inform people about the existence of mHealth apps, invite them to download, encourage them to use the apps, and maintain engagement. This is consistent with an integrative review of methods used to promote mobile apps, which also cited app store optimization via keywords and the inclusion of screenshots and videos for greater conversion rate, the use of push notifications, the promotion of apps via influencers, and the leveraging of user review and ratings [13].

Social media attracted many downloads over a short period, whereas emails were most often used for sharing instructions on how to download apps and interact with research teams for technical support.

The strategies used to promote mHealth apps included paid and unpaid marketing, and metrics such as CPC and CTR were used to measure effectiveness. Offering incentives to people to download and use the app did increase app downloads and use. However, it has been shown that people are less likely to keep using an app after incentivization, although the monetary value of the incentive could have a significant effect on the adherence [3]. Furthermore, a focused strategy is required to maintain a low app churn rate [12].

### Factors Influencing Engagement, Onboarding, or Adherence to mHealth Apps

#### Reasons for User Engagement, Onboarding, or Adherence to the App

Onboarding was often reliant on the ease of downloading and sufficient motivation. In the study by Hui et al [30], adherence stemmed from awareness that a health care professional and a researcher were interested in the results and that using an app to support self-management could improve a participant’s control of their health condition. Additional factors in engagement included the perceptions of the advantages and disadvantages of using apps to support self-management specific to an individual’s health needs, the relevance of the app to the user, the quality of the app, and the behavior change techniques used to promote health [28]. All these factors are among those reported by a literature review that identified retention factors related to apps, such as feedback, appropriate reminders, and in-app support from peers or coaches [33]. Our findings also align with those of another systematic review that listed individualized reminders, user friendliness and technical stability of the apps, and personal support from health care professionals as intervention-related factors influencing adherence [3].

Push notifications and weekly SMS text messages inviting check-in were also used to influence user engagement [26], herein defined by number of check-ins. This is also consistent with the findings from a systematic review [34] that assessed 15 commercial apps for diabetes prevention and found that the app that included the notification features for activity tips, goal progress tips, goals adjustment, and completed goals had the highest engagement mean score (4.5 points out of 5). However, we argue that push notifications should not be a one-size-fits-all solution because marketing research suggests that among app users, just 50% accept push notifications from their favorite app, and 30% disable all push notifications [35].

In our review, user engagement was also influenced by the health goals that participants selected. This finding also aligns with that of a previous systematic review that further suggested that users could disengage at any time and re-engage at a later stage when needed. Thus, this feature might be particularly useful for addiction research targeting relapse prevention strategies [36].

#### Barriers to User Engagement, Onboarding, or Adherence to the App

From the studies included in this review, reasons for nonadoption included problems in app installation [24,30]; the use of other health apps that better suited participants’ needs and preferences; and other concerns, such as prioritizing COVID-19 over the condition addressed by the app [28]. Indeed, marketing can be influenced by competing health information targeting the same users. When people were concerned about contracting COVID-19 and seeking a pandemic-related app, they were less motivated to use an app being promoted to tackle other health issues [17,24]. This could lead to competition among mHealth apps for potential users or health care providers.

Other potential barriers to adoption included a lack of early active engagement with participants to verify that they could install the app, no direct contact with health care professionals, and not enough interactive features. Indeed, as reported in another systematic review, being less informative and less interactive can lead to a very low engagement mean score [34].

The study by Roberts et al [28] reported that technical issues and concerns about data security reduced engagement. Similar concerns about data security with mHealth apps had been previously pointed out in the literature [37,38].

### Return on Investment in Marketing

Although dependent upon the budget available, a decision threshold for the cost of marketing campaigns for mHealth apps to reach a certain level of engagement would be beneficial. According to a study on industry-specific Google benchmarks, for the health care sector overall, these costs amount to a CTR of 3.27% and a CPC of US $2.62 for Google Search and a CTR of 0.59% and a CPC of US $0.63 for the Google Display Network [39]. A similar study on industry-specific Facebook benchmarks produced a CTR of 0.83% and a CPC of US $1.32 for health care [40]. However, it is important to note that comparing CTR and CPC with industry-specific benchmarks for the entire health and medical field should not be the sole method of evaluating effectiveness because these benchmarks may be too broad [17].

### People Involved in Marketing mHealth Apps

The most productive marketing team is a multitude of satisfied users championing the app on social media, encouraging their friends and colleagues to download it, and giving it 5-star reviews. Creating a strong user support system and feedback loop, regularly updating the app based on user feedback, and doing whatever it takes to keep users happy are the most important marketing tactics that can be deployed [12].

We found that in studies that investigated age and sex differences in engagement with apps, the age of research participants did not predict app engagement [24,25]. However, there were statistically significant differences in sex and app use, with more male participants using the apps in question than female participants, but not in the duration of app use [24,30]. This result is contradictory with that of another study that found that female sex positively influenced adherence [3]. Therefore, we could not draw conclusions on the effect of sex and age on app adherence and thus leave it to further research.

Marketers of mHealth apps should always consider the motivations of the app audience; for example, the study by Roberts et al [28] stated that apps promoting walking can be appealing to survivors of cancer. Similar findings were highlighted elsewhere [3]. In addition, consideration must be given to the timing of interventions intended to maintain engagement. Data should be collected to predict the moments that users will be available and receptive to in-app notifications.

### Limitations

We have noted confusion surrounding the term *user engagement*. Engagement with digital health interventions and engagement with mHealth apps are not clearly separated. This study focuses on the latter, defined as a set of actions by a user within an mHealth app [18]. This differs from user engagement with digital health interventions, which is conceptualized in terms of both experience and behavior [41]. This confusion may explain why many of the studies identified during the database searches had to be excluded: they addressed outcomes related to changes in health behavior; for example, most of the excluded papers reported levels of user engagement with a health intervention, such as increased physical activity, but not engagement with an mHealth app. However, we recognize that these definitions are interlinked: research has shown that app engagement can motivate behavior change [25]. We have noted the same confusion with the term *user embedment*. In the 10 reviewed studies, only 1 (10%) referred to embedment as integrating a functionality within the app.

Most of the studies we reviewed (9/10, 90%) were conducted in high-income countries, with half being carried out in the United States (5/10, 50%). This may limit generalizability in low- and middle-income countries. Moreover, we only searched for papers written in French and English. We also note that the filters we applied with our search terms combination to avoid noise could have excluded some potentially useful papers. This could explain why the reviewed studies mostly reported research conducted in the United States and Europe (8/10, 80%).

Our findings may also be subject to observer bias [42] because in every reviewed study the research team members were involved in the diffusion processes. In some of the studies (2/10, 20%), participants received in-person physical assistance with app installation. This would be impossible for users in many settings. The generalizability of these findings is also limited by the fact that none of the included studies covered the dissemination of mHealth apps among health care personnel.

### Implications and Future Research

#### Integration of mHealth Apps Into Routine Clinical Practice

None of the studies we reviewed aimed to address the embedment of mHealth apps in routine practice as part of their marketing strategy. To tackle the issue of the embedment of mHealth apps, researchers have proposed a framework for prescribing apps and outlined the key issues that need to be addressed to enable app dissemination in clinical care. This includes education and awareness, the creation of digital formularies, workflow and electronic health record integration, payment models, and patient or provider support [43]. As suggested by this framework, a starting point for the integration of mHealth apps into routine clinical practice would be education and awareness, meaning the promotion of mHealth apps, the aim of which would be to create a base of users downloading the apps because the number of app downloads and interactions over time also provides an indication of sustained uptake over time [11].

At this critical point of creating a user base, inspired by the synthesis of our findings, we offer a set of recommended uses of different channels (Table 4). This would first be applicable during the launching phase of the app and to lead users through their app onboarding stage. The use of these channels could change depending upon the objective of the marketing strategy.

**Table 4:** Recommended uses of channels to promote mobile health apps.

Channel and recommended use			Targeted people		Supportive marketing concepts
**Email**					
	Inform about the existence of the app, link for download, functionalities, app release notes and app updates, and general information regarding the app (developer, brand owner, and sponsor)	High-level users (influencers and decision-makers) and target base users (health care providers and day-to-day users of the app)		A/B testing	
	Give instructions on how to download and install and use the app	High-level users (influencers and decision-makers) and target base users (health care providers and day-to-day users of the app)		A/B testing	
	Provide technical support and answers to users’ questions; share user guide and tips	Effective users		A/B testing	
**Social media**					
	Inform about the existence of the app, and share the link for download	Potential target base users (health care providers and day-to-day users of the app)		A/B testing, incentivization, loyalty marketing, thought-leadership marketing, and content marketing	
	Engage in direct interactions to provide technical support and answers to users’ questions	Potential target base users (health care providers and day-to-day users of the app)		A/B testing, incentivization, loyalty marketing, thought-leadership marketing, and content marketing	
**Television**					
	Short promotional video report on the app and its functionalities, as well as 1- to 3-minute video spots with speech by high-level users (influencers and decision makers) recommending the apps	Potential target base users (health care users and day-to-day users of the app)		Incentivization and thought-leadership marketing	
**Posters or flyers**					
	Infographics and key text message to inform about the existence of the app and its main value; include a QR code and text to indicate link to download	Potential target base users (health care providers and day-to-day users of the app)		Predictive marketing and incentivization	
**Face-to-face interaction: in-person training or meeting**					
	Inform about the existence of the app, link for download, functionalities, app release notes and app updates, and general information regarding the app (developer, brand owner, and sponsor)	High-level users (influencers and decision makers) and potential target base users (health care providers and day-to-day users of the app)		A/B testing	
	Give instructions on how to download, install, or use the app; share and explain user guide and discuss tips	High-level users (influencers and decision makers) and potential target base users (health care providers and day-to-day users of the app)		A/B testing	
**Face-to-face interaction: in-person ad hoc (unplanned) encounter**					
	Engage in direct interactions to provide technical support and answers to users’ questions	Health care providers and day-to-day users of the app		Remarketing or retargeting and personalization	
	Sell the app (highlight its main value) and manage to install it on users’ devices; explain how to use it and discuss tips	Health care providers and day-to-day users of the app		Remarketing or retargeting and personalization	

Social media could be a beneficial entry point for motivating people to download an app, and human interaction is key during the engagement phase. Therefore, mHealth app promoters should provide users with training and support to start and continue using the apps. This can be done by maintaining communication through social media, including app-dedicated pages. At this point, content marketing—producing content that potential customers find useful—is valuable. It has been demonstrated that content marketing is highly effective in building a loyal user base and converting leads into customers [12].

It is important to note that although social media marketing also tends to attract people who are not the intended audience, communication through email requires a list of targeted email addresses. This entails contacting people directly and requesting their addresses or interacting with someone who will reveal potential users’ email addresses or share an app link with potential users; for instance, a hospital director may share information about an app with hospital staff or share an attendance list containing the email addresses of hospital staff.

Future public health campaigns targeting the parents of young children should consider crafting marketing messages for social media campaigns with animated GIF images as well as images of young children.

As time-varying push notifications have been shown to contribute to mHealth app user engagement, developers should interact with health care providers to implement this strategy. One approach to this is to apply mobile app attribution: the process of recording and measuring the actions of app users, such as installs, level completions, and in-app purchases [12].

#### Future Research

Finally, future research could be dedicated to developing a framework on how to disseminate mHealth apps. Such a framework, in addition to various marketing concepts presented in this review, should take into account additional considerations that are specific to mHealth apps, such as data confidentiality and privacy, and segment users on the marketing funnel [12] based on the best available evidence on engaging users with mHealth apps. One issue impeding the dissemination of apps that emerged in our study was the existence of competing apps. Some researchers have suggested that digital formularies or app libraries could help to address this. Digital formularies provide a short list of available apps, and providers could search these formularies and know what is available for a specific diagnosis or purpose [43]. Further research could explore and expand on the effectiveness of digital formularies as a dissemination channel for mHealth apps and the enablers of embedment of mHealth apps into routine practice. Finally, further research could aim to address the gap in identifying specific marketing strategies that would effectively drive the embedment of mHealth apps into routine practice.

### Conclusions

The dissemination of mHealth apps takes place via face-to-face interactions, email, and printed posters and social media channels with diverse results. The effects of these strategies are reflected in download numbers and user engagement with mHealth apps. The results of this study will serve to guide future research and guide the marketing of mHealth apps for their routine use within the health sector.

The development of a framework for health care designers to promote their apps within health systems would be immensely beneficial. Such a framework would help systematize the dissemination of mHealth apps and guide the impact assessment of the dissemination strategies.

## Appendix

Multimedia Appendix 1 PRISMA (Preferred Reporting Items for Systematic Reviews and Meta-Analyses) checklist.

Multimedia Appendix 2 Search strategy.

## Abbreviations

CPC: cost per click
CTR: clickthrough rate
GIF: graphic interchange format
mHealth: mobile health
PRISMA: Preferred Reporting Items for Systematic Reviews and Meta-Analyses
SIGN: Scottish Intercollegiate Guidelines Network
UAC: Universal App Campaigns
