# Supplementary material for: Dissemination Strategies for mHealth Apps: Systematic Review
Source: JMIR Mhealth Uhealth. 2024 Jan 5;12:e50293. doi: 10.2196/50293 (PMC10799285; doi:10.2196/50293)
Supplement: Multimedia Appendix 2 [file mhealth_v12i1e50293_app2.doc]

### **Dissemination of mHealth Applications: A Systematic Review**

### **Search strategy**

### **PubMed**

(dissemination[TIAB] OR marketing[TIAB] OR promotion[TIAB] OR share[TIAB] OR sharing[TIAB] OR diffusion[TIAB]) AND (mhealth[TIAB] OR “mobile health”) AND (app[TIAB] OR apps[TIAB] OR application[TIAB])

Filters applied in Pubmed:

- Period: Last 5 years (2018-2022).
- Article type: Evaluation study, Observational study, clinical study, clinical trial (phase I, II, III), controlled clinical trials, randomized clinical trial, Meta-analysis.
- Species: Human.
- Languages: English, French

### **Scopus**

TITLE-ABS (dissemination OR marketing OR promotion OR share OR sharing OR diffusion ) AND TITLE-ABS ( mhealth OR "mobile health" ) AND TITLE-ABS ( app OR apps OR application ) AND PUBYEAR > 2017 AND SUBJAREA ( medi ) AND ( LIMIT-TO ( DOCTYPE , "ar" ) ) AND ( LIMIT-TO ( LANGUAGE , "English" ) OR LIMIT-TO ( LANGUAGE , "French" ) )

Filters applied in Scopus:

- Period: Last 5 years (2018-2022).
- Subject area: medicine
- Document type: article
- Languages: English, French

### **CINAHL Complete**

ABS ( dissemination OR marketing OR promotion OR share OR sharing OR diffusion ) AND ABS ( mhealth OR "mobile health" ) AND ABS ( app OR apps OR application )

Filters in CINAHL Complete:

- Period: Last 5 years (2018-2022).
- Article type: Evaluation study, Observational study, clinical study, clinical trial (phase I, II, III, IV), controlled clinical trials, randomized clinical trial,
- Languages: English, French

### **PsycInfo**

TIAB(dissemination OR marketing OR promotion OR share OR sharing OR diffusion) AND TIAB(mhealth OR "mobile health") AND TIAB(app OR apps OR application)

Filters applied in PsycInfo:

- Date: After 31 December 2017
- Methodology filter: Clinical Case Study, Clinical Trial, Empirical Study, Field Study, Followup Study, Longitudinal Study, Meta Analysis, Metasynthesis, Nonclinical Case Study, Prospective Study, Qualitative Study, Quantitative Study, Retrospective Study
- Language filter: English, French
- Population filter: Human

The terms used in databases search were:

- dissemination
- marketing
- promotion
- share
- sharing
- diffusion
- mhealth
- mobile health
- app
- apps
- application
